# Acute Pancreatitis Associated With Blinatumomab Treatment in a Patient With Acute Lymphoblastic Leukemia

**DOI:** 10.7759/cureus.82803

**Published:** 2025-04-22

**Authors:** Ammaar Y Kazi, Zuhair Zaidi, Mohammad Zahid

**Affiliations:** 1 Department of Hematology and Oncology, University of Texas (UT) Southwestern Medical School, Dallas, USA

**Keywords:** acute pancreatitis, b-all, blinatumomab, cytokine release syndrome, immunotherapy

## Abstract

Blinatumomab, a bispecific T-cell engager, has significantly advanced the treatment of relapsed and refractory B-cell acute lymphoblastic leukemia (B-ALL), with well-established toxicities such as cytokine release syndrome and neurotoxicity. However, pancreatitis remains an exceptionally rare and underrecognized adverse effect, with limited reports outside of clinical trials. This case report presents one of the first documented instances of acute pancreatitis in a patient receiving blinatumomab in a real-world setting, diagnosed by elevated serum lipase and confirmatory CT imaging. The patient’s presentation and resolution with supportive care underscore the importance of promptly recognizing and managing this potential complication. Further investigation is warranted to elucidate the pathophysiological mechanisms of this association and to guide clinical decisions regarding blinatumomab safety and rechallenge protocols in affected patients.

## Introduction

B-cell acute lymphoblastic leukemia (B-ALL) is a malignant proliferation of B-lymphoid progenitors, with cure rates up to 90% in the pediatric age group. Unfortunately, outcomes in adolescents and adults are inferior, with cure rates of only about 40% and a progressive decline in prognosis with age [1,2]. Blinatumomab is a novel bispecific T-cell engager antibody construct that binds to cluster of differentiation 19 (CD19), a transmembrane protein expressed on B cells, and cluster of differentiation 3 (CD3), a component of the T-cell receptor complex on cytotoxic T lymphocytes. This dual binding facilitates the formation of a cytolytic synapse, resulting in targeted cellular cytotoxicity of B cells, including malignant B-lymphoblasts. Blinatumomab is approved for use in measurable residual disease (MRD)-positive, relapsed/refractory (R/R) Philadelphia chromosome-negative (Ph(-)) B-ALL, and Philadelphia chromosome-positive (Ph(+)) B-ALL, representing an advanced therapeutic strategy with expanding roles in both frontline and consolidation settings [1,3-5].

Despite its effectiveness, the administration of blinatumomab has been associated with adverse effects, the most notable of which include cytokine release syndrome (CRS) and immune effector cell-associated neurotoxicity syndrome (ICANS), along with neutropenia, thrombocytopenia, and hypogammaglobulinemia. Pancreatitis is a serious, albeit less common and underrecognized, adverse effect associated with blinatumomab [6]. Initial concerns were raised by Health Canada in 2016 following review of clinical trial data and post-marketing surveillance reports, including cases of pancreatitis, one of which was fatal [7]. This association was further examined in the TOWER trial, a phase 3 study comparing blinatumomab to standard chemotherapy in R/R B-ALL patients [3]. Among the 267 participants treated with blinatumomab, one case (0.4%) of acute pancreatitis was potentially attributable to the drug. Moreover, analysis of the FDA Adverse Event Reporting System (FAERS) from U.S. market approval through September 2016 identified five cases of pancreatitis among 580 treated patients (0.9%), reinforcing the need for vigilance and careful assessment of pancreatic function in patients receiving this therapy [6].

We report the case of a young male patient with R/R B-ALL treated with salvage blinatumomab, whose course was complicated by acute pancreatitis.

## Case presentation

A young male patient in his 30s was diagnosed with (Ph(-)) B-ALL at an outside hospital. He received induction chemotherapy using Cancer and Leukemia Group B (CALGB) 10403 Course I without asparaginase due to institutional limitations, achieving morphologic remission by the end of induction (MRD status was not assessed). He proceeded with consolidation therapy using the hyperfractionated cyclophosphamide, vincristine, doxorubicin, and dexamethasone (hyperCVAD) regimen for eight cycles and remained in morphologic remission. Two months after completing consolidation, he experienced disease relapse and transferred to our center. Bone marrow biopsy revealed 90% lymphoblasts with diploid cytogenetics. Next-generation sequencing identified a neuroblastoma RAS viral oncogene homolog (NRAS) G13D mutation and homozygous deletion of cyclin-dependent kinase inhibitor 2A (CDKN2A) and cyclin-dependent kinase inhibitor 2B (CDKN2B). He began salvage therapy with mini-hyperCVD (a modified version of hyperCVAD omitting anthracyclines) in combination with inotuzumab ozogamicin. However, a follow-up bone marrow biopsy after two cycles showed persistent disease with 15% lymphoblasts.

After review of his disease course and resistant disease, the patient expressed his goals to continue treatment to prolong life, and was started on salvage treatment with blinatumomab. Blinatumomab was initiated at a lower dose of 9 mcg/day for seven days. His course was complicated by grade 1 CRS when he developed a fever (maximum temperature 101.9 °F), which was managed with supportive care. He did not develop any respiratory distress, hypotension, or ICANS. The blinatumomab dose was increased to 28 mcg/day, and the patient was discharged to complete 28 days of continuous infusion. The patient continued intrathecal chemotherapy, alternating with 12 mg of methotrexate and 100 mg of cytarabine as part of central nervous system-directed therapy for his Ph(-) B-ALL (he received cytarabine on day 34 of his cycle). 

**Figure 1:**
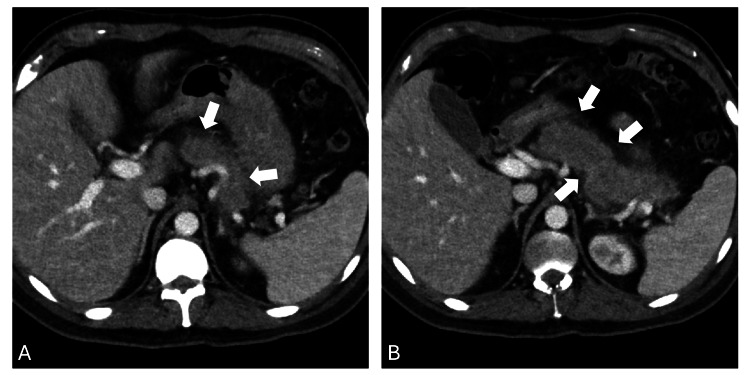
Edematous pancreas with peripancreatic fat stranding consistent with pancreatitis Panel A: Axial contrast-enhanced CT image showing an enlarged, edematous pancreas (arrow) with surrounding peripancreatic fat stranding, characteristic of acute interstitial pancreatitis. No evidence of necrosis or fluid collections is present. Panel B: Axial CT demonstrating peripancreatic fat stranding (arrow) and preserved pancreatic enhancement without ductal dilatation or pseudocyst formation, consistent with uncomplicated acute pancreatitis.

The patient tolerated the medication well up until day 35 (one week after finishing his 28-day course), when he started complaining of severe, dull, epigastric abdominal pain. The pain radiated to the back, and it gradually increased in intensity, becoming sharper in nature over the course of three days, at which time he presented to the emergency room. Serum lipase levels were elevated at 1177 units/L (normal: 7-59). Computed tomography (CT) imaging of the abdomen/pelvis showed an edematous pancreas with peri-pancreatic fat stranding (Figure 1). A diagnosis of acute pancreatitis was made, and the patient was admitted and treated with intravenous fluids, pain control, and antibiotics. He denied any recent alcohol use, abdominal trauma, or new medications. Triglycerides were mildly elevated at 266 mg/dL (normal: 50-150 mg/dL), and no cholelithiasis was noted on abdominal imaging. Blinatumomab was suspected to be the most likely etiology of pancreatitis, and it was permanently discontinued. After three days, clinical improvement was noted, and his diet was advanced as he tolerated more oral intake. He was discharged after a week of hospitalization with complete normalization of his serum lipase levels.

## Discussion

The current case elaborates the clinical course of a young patient with R/R B-ALL who developed acute pancreatitis attributed to blinatumomab. Initial concerns for a potential association between acute pancreatitis and blinatumomab were raised by Health Canada in 2016 following a review of clinical trial data and post-marketing surveillance reports, which included four instances of pancreatitis, one of which was fatal [7]. Analysis of the TOWER trial, a phase 3 study comparing blinatumomab to standard chemotherapy in 267 R/R B-ALL patients, reported one (0.4%) case of acute pancreatitis, which was attributed to blinatumomab [3]. Moreover, an examination of the FAERS database from the U.S. marketing approval date to September 2016 identified five cases of pancreatitis associated with blinatumomab out of 580 treated patients (0.9%) [8].

In general, pancreatitis is not a frequently encountered toxicity associated with immunotherapy agents. Michot et al. indicated a 2.3% incidence of immune-related asymptomatic lipase elevation and a 0.3% overall incidence of clinically symptomatic immune-related pancreatitis due to immune checkpoint inhibitors (ICI) [8]. A meta-analysis by George et al. reported that although no fatalities from pancreatitis occurred with the use of ICIs, the occurrence of severe pancreatitis stood at ~1% [9].

The mechanism underlying pancreatic injury from blinatumomab is unclear. Extrapolating from reports of pancreatitis attributed to ICIs, one theorized mechanism is CRS, where the robust polyclonal activation of T-cells by blinatumomab leads to an excessive release of cytokines such as interleukin-6 (IL-6), tumor necrosis factor-alpha (TNF-α), and interferon-gamma (IFN-γ), provoking a systemic inflammatory response, causing direct injury to pancreatic tissue, possible reduction in blood flow, and local tissue hypoxia [10].

The prompt institution of pain control, intravenous hydration, nutritional support, antibiotics (if applicable), and removal of the offending agent is paramount to achieving a favorable outcome. Management of immune-related pancreatitis is nuanced and follows specific guidelines. The National Comprehensive Cancer Network (NCCN) Guidelines advise against intervening in cases where patients exhibit asymptomatic elevations in pancreatic enzymes. For symptomatic patients, however, the continuation of immunotherapy is contingent on the careful monitoring of these enzymes. In instances of severe acute pancreatitis, systemic steroids are considered with a slow taper, and permanent discontinuation of ICI therapy is recommended. Owing to the severity of pancreatitis, blinatumomab was permanently discontinued in our patient. 

## Conclusions

In conclusion, blinatumomab is a highly effective immunotherapy for R/R B-ALL, and its widespread use is increasing, with emerging evidence supporting its efficacy even in the frontline setting. While oncology providers are well-versed in diagnosing and managing CRS and ICANS, the most common toxicities associated with this agent, acute pancreatitis, represents a rare, underrecognized, but potentially serious toxic effect. Acute epigastric abdominal pain in a patient receiving blinatumomab should prompt suspicion and evaluation with serum lipase and amylase and consideration of CT imaging in cases of severe pain. Though pancreatitis has been documented in post-marketing surveillance, it remains rarely reported in the medical literature, reinforcing the need for awareness and pancreatic monitoring in patients receiving this drug. Interruption of blinatumomab is warranted when pancreatitis occurs. However, it remains unclear whether permanent discontinuation is necessary or if rechallenging is appropriate, particularly in patients who recover rapidly or who exhibit isolated enzyme elevation without symptoms or imaging findings.
